# CHIASM-Net: Artificial Intelligence-Based Direct Identification of Chiasmal Abnormalities in Albinism

**DOI:** 10.1167/iovs.64.13.14

**Published:** 2023-10-10

**Authors:** Robert J. Puzniak, Gokulraj T. Prabhakaran, Rebecca J. McLean, Sebastian Stober, Sarim Ather, Frank A. Proudlock, Irene Gottlob, Robert A. Dineen, Michael B. Hoffmann

**Affiliations:** 1 Visual Processing Lab, Department of Ophthalmology, Otto-von-Guericke-University, Magdeburg, Germany; 2 Department of Neuroscience, Psychology, and Behaviour, University of Leicester, Leicester, United Kingdom; 3 University of Leicester Ulverscroft Eye Unit, University of Leicester, Leicester Royal Infirmary, Leicester, United Kingdom; 4 Artificial Intelligence Lab, Institute for Intelligent Cooperating Systems, Otto-von-Guericke-University, Magdeburg, Germany; 5 Department of Radiology, John Radcliffe Hospital, Oxford University Hospitals NHS Foundation Trust, Headington, Oxford, United Kingdom; 6 Cooper Neurological Institute and Cooper Medical School of Rowan University, Camden, New Jersey, United States; 7 Mental Health and Clinical Neuroscience, Faculty of Medicine and Health Sciences, University of Nottingham, Nottingham, United Kingdom; 8 NIHR Nottingham Biomedical Research Centre, Nottingham, United Kingdom; 9 Center for Behavioral Brain Sciences, Otto-von-Guericke-Universität, Magdeburg, Germany

**Keywords:** albinism, optic chiasm, artificial intelligence (AI), neuroimaging, chiasmal malformations

## Abstract

**Purpose:**

Albinism is a congenital disorder affecting pigmentation levels, structure, and function of the visual system. The identification of anatomical changes typical for people with albinism (PWA), such as optic chiasm malformations, could become an important component of diagnostics. Here, we tested an application of convolutional neural networks (CNNs) for this purpose.

**Methods:**

We established and evaluated a CNN, referred to as CHIASM-Net, for the detection of chiasmal malformations from anatomic magnetic resonance (MR) images of the brain. CHIASM-Net, composed of encoding and classification modules, was developed using MR images of controls (*n* = 1708) and PWA (*n* = 32). Evaluation involved 8-fold cross validation involving accuracy, precision, recall, and F1-score metrics and was performed on a subset of controls and PWA samples excluded from the training. In addition to quantitative metrics, we used Explainable AI (XAI) methods that granted insights into factors driving the predictions of CHIASM-Net.

**Results:**

The results for the scenario indicated an accuracy of 85 ± 14%, precision of 90 ± 14% and recall of 81 ± 18%. XAI methods revealed that the predictions of CHIASM-Net are driven by optic-chiasm white matter and by the optic tracts.

**Conclusions:**

CHIASM-Net was demonstrated to use relevant regions of the optic chiasm for albinism detection from magnetic resonance imaging (MRI) brain anatomies. This indicates the strong potential of CNN-based approaches for visual pathway analysis and ultimately diagnostics.

Albinism is a rare congenital disorder that affects pigmentation levels,^1^^,^^2^ as well as the development and organization of human visual system.^3^ The changes in the visual system include chiasmal misrouting, that is, enhanced nerve crossing at the chiasm. The detection of chiasmal misrouting is a relevant criterion for albinism diagnosis, particularly in individuals with mild or absent skin and hair involvement.^4^ Currently, optic nerve misrouting at the chiasm is assessed using visually evoked potentials (VEPs),^5^^,^^6^ which requires patient cooperation and can be affected by the degree of visual impairment.^6^ Recognition of these limitations prompted the development of alternative methods for a direct, anatomy-based detection of optic nerve misrouting at the chiasm.^7^

The first wave of methods for anatomy-based detection of optic nerve misrouting relied on diffusion magnetic resonance imaging (dMRI),^8^ which is based on the detection of the diffusion of water molecules in the tissue. In addition to quantifying microstructural properties of tissues,^9^ dMRI can be also used for tractography,^10^ that is, reconstruction of neural pathways from the optic nerves to the visual cortex and beyond.^11^ Given this utility, dMRI was expected to capture and indicate an excessive proportion of crossing nerves in the chiasm in people with albinism (PWA), as opposed to the normal ratio of 53% in healthy controls.^12^ Indeed, dMRI captured differences in nerve crossing in the chiasm between healthy controls and PWA at the group level,^13^ and with further improvements, indicated potential for robust albinism diagnostics at the individual level.^14^ The dMRI-based diagnostics, however, are limited by considerable data acquisition time (approximately 45 minutes as for the protocol used by Puzniak et al.^14^) and demanding data analysis, restricting its applicability in clinical routine.

In consideration of these limitations of economically and technically expensive dMRI data, we have previously used an alternative approach for the detection of chiasmal misrouting using the clinically prevalent T1-weighted (T1w) images, which reflects the spatial location of different tissue types (e.g. white matter and grey matter).^15^ The rationale behind this approach was to leverage on potential structural abnormalities of the optic chiasm induced by nerve misrouting. In particular, earlier morphological studies of the chiasm in PWA revealed that albinism is characterized by reduced width of the optic nerves and the optic chiasm,^16^^–^^18^ with Schmitz et al. additionally reporting wider angles between optic tracts for PWA. The importance of these features for diagnostics of misrouting in the chiasm is still unclear, primarily due to (1) conflicting studies reporting no significant differences in the chiasm's morphology between healthy controls and PWA,^19^ and (2) the lack of attempts to classify healthy controls and PWA based on the reported distinguishing chiasm features. Recently, however, this uncharted area for the detection of chiasmal malformations from T1w images has been investigated with a deep learning (DL) approach.^15^ Specifically, it was demonstrated that a convolutional neural network (CNN), trained on control data for segmentation of the chiasm, underperforms when applied to T1w brain images of PWA. This confirmed that the different structural configuration of the optic chiasm in healthy controls versus PWA can be utilized for diagnostics via DL-based approaches. At the same time, the study did not investigate the specific nature of the features distinguishing normal from abnormal chiasms, nor did it attempt to use classifier CNNs to assess the potential of applying DL-based methods for the detection of chiasmal malformations from T1w images.

In the present study, we aim to fill this gap by: (a) developing the first classification CNN to detect the presence of chiasmal malformations from T1w images, and (b) evaluating the CNN's performance quantitatively and qualitatively. The quantitative evaluation involves the assessment of performance of the established CNN on unseen data of PWA and healthy controls. Our findings are expected to provide important insight into the applicability of artificial intelligence (AI)-methods to the problem of detecting chiasmal malformations from T1w images. The qualitative evaluation involves the application of a range of Explainable AI (XAI)^20^ methods in order to identify the features relevant for outcome prediction. This type of assessment provides additional means to validate the established CNN. Further, it provides valuable insights into the specific anatomic features that distinguish normal and abnormal chiasms, as in PWA.

## Methods

For the above objectives, we established a CNN referred to as CHIASM-Net which uses patches of T1w images, including the human optic chiasm for classification as either normal or albinotic chiasms, depending on the presence of anatomic malformations typical for albinism. The magnetic resonance imaging (MRI) data used in the study originate from publicly available datasets containing T1w images of healthy controls^21^^–^^26^ and PWA^27^ as well as a non-public PWA data set.^13^ For detailed information on the data and its pre-processing pipeline, see “Supplement 1: Data sources” and “Supplement 2: Preprocessing of T1w MRI images.” The general overview of the development of CHIASM-Net is provided in the subsection Implementation, with the detailed information on the identification of the architecture and its training in “Supplement 3: Feature extraction module” and “Supplement 4: Classification module.” The methods for evaluation and validation of the established CHIASM-Net are described in the subsection: Evaluation metrics.

### Implementation

This section describes the primary motivation for the creation of a custom CNN (design), the architecture of the outcome CHIASM-Net (architecture), and the segregation of training and evaluation data (data splits).

#### Design

To reduce the model size and the impact of structures other than optic chiasm on the classification metrics, the CNN was designed to operate on small patches of T1w images (dimensions: 24 × 24 × 8 mm), encompassing the optic chiasm. The size ensured the optic chiasm (average width and height of 15.0 mm and 3.5 mm, respectively^28^) to be completely contained within the patch, even if subjected to data augmentation and transformations, such as translation or rotation. The patches were defined with a combination of techniques (automated, semi-automated, and manual), with each patch receiving a subsequent direct check to ensure a full coverage of the chiasm (see “Supplement 2: Preprocessing of T1w MRI images”). The small input size prevented us from using well-established CNN architectures, designed typically for medical images of larger dimensions. Accordingly, we designed a custom CNN (CHIASM-Net) governed by the classical structure and rules of classifying CNNs.

#### Architecture

CHIASM-Net comprises of two main components. The first one, the feature extraction module, encodes (extracts) the relevant features of the original input and outputs them for further processing. The second component, the classification module, digests the encoding and based on it assigns a score between 0 and 1 (with 0 indicating control and 1 indicating PWA). Supplementary Table S2 provides a detailed overview of the CHIASM-Net architecture. “Supplement 3: Feature extraction module” and “Supplement 4: Classification module” describes the optimization-analyses in the design of CHIASM-Net. The results of those analyses and their consequences for the architecture are presented respectively in “Supplement 6: Results for feature extraction module” and “Supplement 7: Results for classification module.”

#### Data Splits

The details on data splits and training of feature extraction module are provided in “Supplement 3: Feature extraction module.” For the training and evaluation of the classification module of the CHIASM-Net, we split the data into 2 groups for training: (1) the TRAIN group and (2) the DEV_TRAIN group, and 2 groups for evaluation: (1) the TEST1 and (2) the TEST2, which were used in 2 distinct scenarios, respectively.

1. The TRAIN data (1274 controls and 23 PWA; Supplementary Table S3) were used exclusively for training of weights of the classification layer. Importantly, due to a high-class imbalance (i.e. unequal number of control and PWA samples), the samples from the minority dataset (PWA) in the TRAIN data were upsampled by a matching factor (approximately 55) to balance the two existing classes, which allows for stable training.
2. The DEV_TRAIN (216 controls and 4 PWA) data were used to monitor the performance of the network over training and ultimately select the best set of weights for the classification layer. Similar to the TRAIN data, the DEV_TRAIN data was upsampled by an appropriate factor to prevent bias introduced by a class imbalance.
3. The TEST1 (4 controls and 4 PWA) contained an equal number of samples for controls and PWA (with 3 samples in each group coming from the Ather et al. dataset and remaining one from the CHIASM dataset). This allowed for the evaluation of the network's performance on a balanced set of data excluded from training, although acquired from the same source as the training data.
4. The TEST2 (214 controls and 1 PWA) sample emulated the real-life scenario. Although we were not able to simulate the true proportions of albinism to controls (approximately between 1:14000 and 1:20000^4^^,^^29^), we used a single PWA sample from the CHIASM dataset and remaining samples (not involved in training) from all control datasets to obtain a proportion of 1:214. It should be noted that, due to the presence of only a single albinism sample, the results for TEST2 cannot provide a meaningful validation of performance. At the same time, however, a weak performance on TEST2 sample, for example, due to high number of false positive results, would indicate critical shortcoming of proposed approach. For this reason, we decided to include the test on 214 controls and single PWA in the analysis.

The above-described data splits were used exclusively for training of the classification module and are fundamentally different from those used for training of the feature extraction module. Importantly, although both modules were trained on the images of the same individuals, we modified the input images to prevent data leakage. Specifically, the training of the feature extraction module was performed on the images with the exclusion of the optic chiasm, whereas classification was trained on extracted patches with optic chiasm only. For more details on differences between training data for both modules refer to “Supplement 3: Feature extraction module” and “Supplement 4: Classification module.”

Importantly, training and evaluation of the CHIASM-Net with above defined splits (TRAIN, DEV_TRAIN, TEST1, and TEST2) was repeated eight times, as we used an eight-fold validation approach to obtain more robust performance estimates. Specifically, the samples (both controls and PWA) from each dataset were divided into eight equal subsets (except for the CHIASM, which was divided as 9 subsets). Out of those:

• six subsets from each dataset were included in the TRAIN group (resulting in 1274 controls and 23 PWA, as described in (1));
• one subset from each dataset was included in the DEV_TRAIN group (resulting in 216 controls and 4 PWA, as described in (2));
• TEST1 group included the remaining one subset from Ather et al. and one out of two remaining subsets for CHIASM (resulting in 4 controls and 4 PWA, as described in (3));
• TEST2 group included the remaining subset of CHIASM and a remaining single subset from all other datasets (resulting in 216 controls and 1 PWA, as described in (4)).

The selection of k = 8 for k-fold validation was motivated by finding a trade-off between the training sample size (essential due to high variability of optic chiasm malformations in albinism) and the testing set sample size that is sufficient for meaningful conclusions. With eight-fold validation, we effectively used 87.5% of data for training (75% for training and 12.5% for training validation) and the remaining 12.5% for testing. The whole procedure being repeated eight times, enhanced the statistical power calculated across the folds.

Subsequently, we performed the training with TRAIN and DEV_TRAIN groups and evaluated the CHIASM-Net performance using the defined TEST1 and TEST2 groups. By averaging the evaluation metrics from the repetitions, we gained insight into the general capabilities of the proposed CHIASM-Net, which is less dependent on one-time selection of training and test data. For more details on the preparation of the training data, for example, preprocessing and upsampling steps, refer to “Supplement 4: Classification module.”

### Evaluation Metrics

In the presented study, the evaluations were performed at two separate stages. The first stage involved testing a range of autoencoders for creating a feature extraction module. Because the description of the development of this module is provided entirely in the supplementary material, we provide the description of applied metrics in the supplement also (“Supplement 5: Evaluation of the feature extraction module”). The second stage involved the evaluation of the trained classification module. This corresponds to the evaluation of the full network and provides meaningful performance estimates for the established CHIASM-Net. We used two distinct approaches:

#### Quantitative Metrics

The quality of the network predictions was described by a range of quantitative metrics that are standardly used for evaluation of machine learning classifiers. Specifically, given the true positives (TPs), false positives (FPs; type I error), false negatives (FNs; type II error), and true negatives (TNs), they are defined as:

(1) $$\begin{array}{c} \begin{array}{c} Accuracy=\;\frac{TP\;+\;TN}{TP\;+\;FP\;+\;FN\;+\;TN}\\ Precision=\;\frac{TP}{TP\;+\;FP}\\ Recall\;or\;Sensitivity=\;\frac{TP}{TP\;+\;FN}\\ F_{1}=\frac{2}{\frac{1}{Recall}\;+\;\frac{1}{Precision}}=\;\frac{2\;\cdot \;TP}{2\;\cdot \;TP\;+\;FP\;+\;FN} \end{array} \end{array}$$

#### Explainable AI Methods

Importantly, an applied CNN-model can show a strong quantitative performance, although it may not be directly driven by the physiologically plausible and relevant data features, but instead by factors that indirectly or by chance correlate with the relevant features. The resulting model would be error-prone and consequently of no relevance for applications in medical imaging. Therefore, whereas positive quantitative metrics are indispensable, they must be corroborated by feature-identification methods. For this purpose, we applied XAI methods to understand whether the predictions are indeed driven by optic chiasm structure, or other factors present in the image. For instance, intensity of neighboring blood vessels might indirectly indicate presence of malformations. We used a range of XAI method provided by the Captum library,^30^ specifically: saliency maps,^31^ DeepLIFT,^32^ and Occlusion.^33^

#### Supplementary Material

For detailed information see Supplementary Material.^34^^–^^57^

## Results

The results in this section present the evaluation outcome of the fully trained CHIASM-Net.

### Quantitative Metrics

We evaluated the CHIASM-Net performance on two separate testing datasets, TEST1 and TEST2 (see the Methods section). TEST1 comprised an equal number of images of healthy controls and PWA, whereas TEST2 comprised 214 images of healthy controls and a single image of PWA. The evaluation of TEST1 and TEST2 was repeated 8 times for different combinations of training and testing data (8-fold validation). The quantitative metrics, expressed by area under receiver operator characteristic (AUROC), accuracy, precision, and recall, were obtained by taking the average from all eight folds (see Supplementary Fig. S3), and the optimal decision threshold (maximizing the difference between the true positive and false positive rates) was separately calculated for each fold.

For the TEST1 group, we report a good performance of CHIASM-Net with an overall accuracy of 85 ± 14 % (Table 1). The comparatively large standard deviation is presumably attributed to a lower performance in two out of eight folds (see Supplementary Fig. S3).

**Table 1.** CHIASM-Net Performance on the TEST1 and TEST2 Data (Averaged Across All Folds)

	CNN With Fine-Tuned Classifier			
	TEST1		TEST2	
	Mean, %	Standard Dev., %	Mean, %	Standard Dev., %
Accuracy	85	14	100	1
Precision	90	14	84	31
Recall	81	18	100	0
F1-score	85	14	88	25

When expressing the results obtained for TEST1 (composed of 4 healthy controls and 4 PWA) as a confusion matrix (Table 2) we observed on average 3.125 TPs (PWA samples assigned as PWA; 0 corresponds to worst performance, and 4 to best), 3.125 TNs (controls classified as controls), 0.375 FPs (controls classified as PWA), and 0.75 FNs (PWA classified as controls).

**Table 2.** Average of Confusion Matrices For CHIASM-Net Results on TEST1 and TEST2 Data

		Prediction			
		TEST1		TEST2	
		Control	Albinism	Control	Albinism
True	Control	3.125	0.375	209.4	0.6
	Albinism	0.75	3.125	0.0	1.0

The main drawback of the estimations based on the TEST1 sample is the balance of classes (healthy controls and PWA), which is not representative of real-world scenarios. For this reason, we additionally evaluated the model's performance on a second sample TEST2 with the highest class imbalance of 1:≈214 (PWA versus controls, respectively) that could be created with the available data. It is essential to keep in mind that (a) this experiment is a mere approximation of the actual rarity of albinism (approximately between 1:14000 and 1:20000) and (b) its purpose is to attempt to falsify rather than verify the model's performance. In this experiment, the CHIASM-Net achieved perfect recall of 100 ± 0% and precision of 84 ± 31%. Because the measured accuracy of 100 ± 1% is biased due to large number of false samples, the overall performance of the model is better expressed with F1-score, equal to 88 ± 25% (see Table 1). When analyzing the confusion matrix (see Table 2) we observed an average of 209.4 TPs with an average of 0.6 FPs, and an ideal average of 1.0 TN with 0.0 FNs.

### Explanation of Predictions With XAI

In this section, we report the qualitative findings from our investigation to determine whether the network's predictions are driven by apprehensible properties of input (for e.g. voxels belonging to optic chiasm rather than blood vessels).

#### Comparison of Averaged Images for Both Healthy Controls and PWA

**Figure 1.**
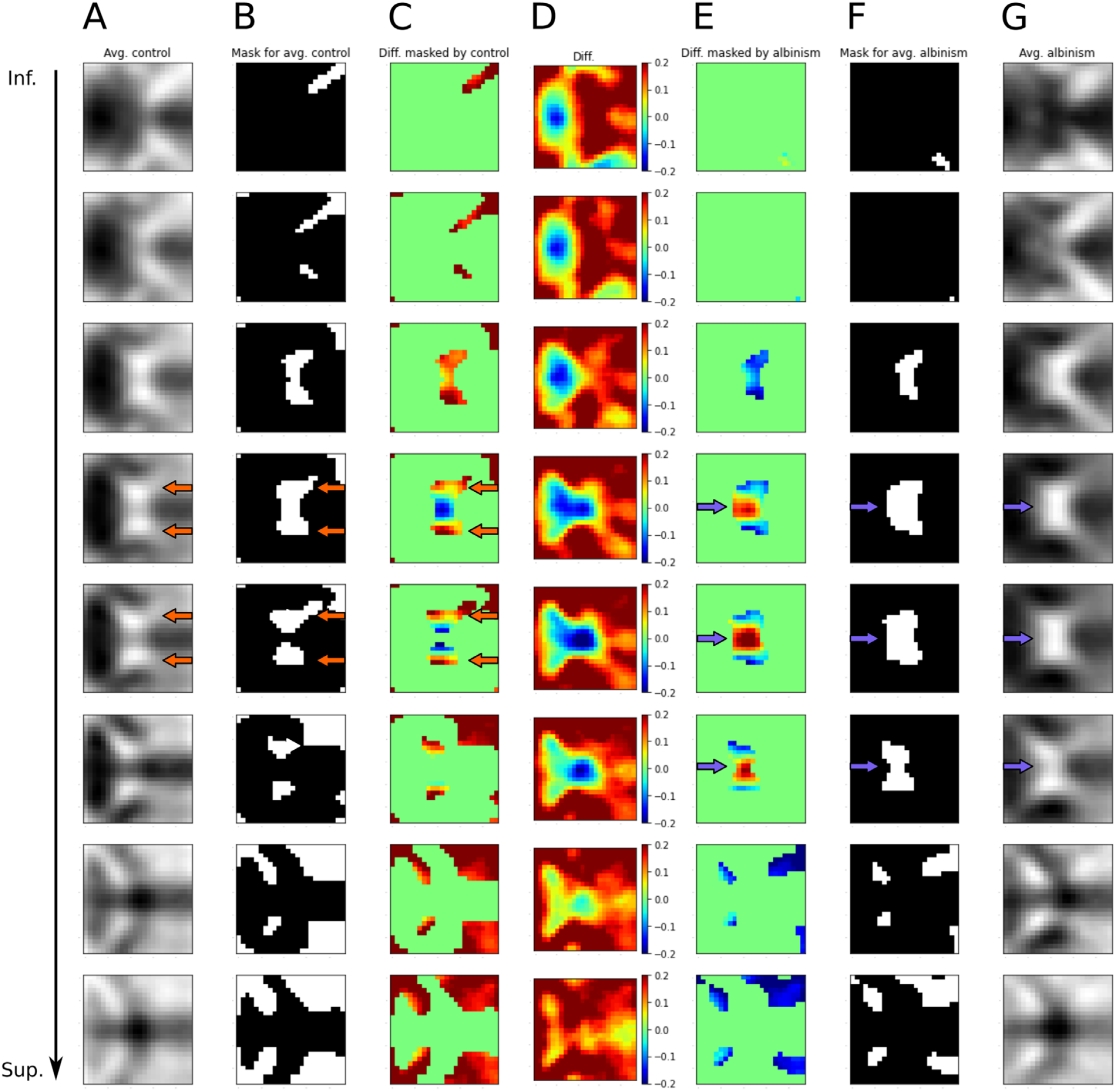
**Differences between averaged images of controls and albinism from the TRAIN dataset from the fold 5.** (**A**) Slices from averaged images of controls. (**B**) Binary mask of voxels (intensity threshold: <0.75) from the averaged control image. (**C**) Difference between averaged control and albinism images masked by the binarized image **B**. (**D**) Difference between averaged control and albinism images. (**E**) Difference between averaged albinism and controls images masked by the binarized image **F** (note the inverted intensity as compared to columns **C** and **D**). (**F**) Binary mask of voxels (intensity threshold: <0.6) from the averaged albinism image. (**G**) Averaged albinism images. All presented images use fixed heatmap with constant range of values. Images for albinism demonstrate higher intensity in the central parts of chiasm (*blue arrows*) as compared to healthy controls (column **E**), which indicates a more compact structure. In contrast, controls show higher intensities on the lateral parts of optic tracts and the chiasm (*red arrows*), which indicates an increased width of the latter (column **C**).

Before inspecting the decision making process behind CHIASM-Net's predictions, we first analyzed the general properties of the input. For this purpose, we calculated two averaged input images for both classes (healthy controls and PWA) and computed the difference between them (Fig. 1). Visual inspection of the average image “control – PWA” revealed higher intensities of voxels on the lateral parts of the chiasm and optic tracts. In contrast, for “PWA – control” the highest voxel intensity values were observed in the central parts of the optic chiasm and the medial parts of the optic nerves.

#### Features of Averaged Images That Drive CNN Predictions

**Figure 2.**
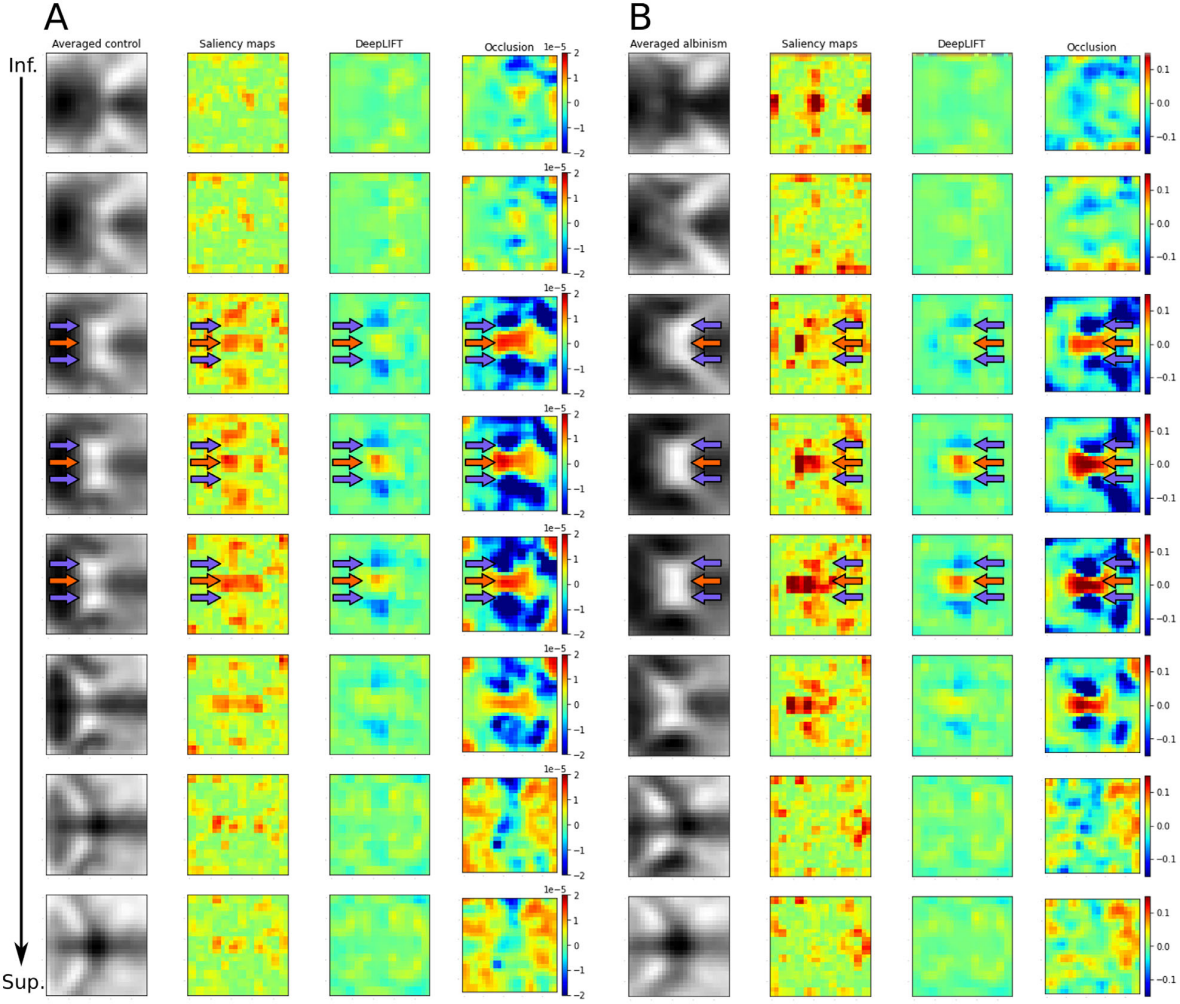
**Inspection of predicting process of CHIASM-Net using image of averaged controls** (**A**) **and albinism** (**B**)**.** The first column demonstrates subsequent averaged slices of control (**A**) and albinism (**B**), from inferior to superior slice. The following three columns indicate the regions of input driving the predictions, as assessed with, from left to right, saliency maps, DeepLIFT, and Occlusion methods. For “Saliency maps” and “DeepLIFT” hot colors mark areas where high voxel intensities positively contribute to the prediction of albinism. In the case of “Occlusion,” warmer colors indicate areas that contribute strongly to the outcome predictions. It should be noted that panels (**A**) and (**B**) use different value ranges. All XAI methods consistently indicate that higher intensities of central voxels of the optic chiasm positively contribute to the prediction of albinism (*red arrows*), whereas the higher intensity in the lateral parts of the optic chiasm has the opposite effect (*blue arrows*).

In the next step, we performed an experiment where we provided the averaged images of controls and albinism as the input to CHIASM-Net. Subsequently, we applied several XAI methods to identify which parts of the input most contribute to the outcome score (Fig. 2).

All methods consistently indicated that high values in the central part of the image (which corresponds to the center of optic chiasm) positively contribute to the prediction of albinism (see Fig. 2). At the same time, higher intensities in the outer parts of the chiasm negatively contributed to the prediction of albinism, thus positively contribute to the prediction of control (see Fig. 2). This matches the patterns present in the training data, as shown in Figure 1.

#### Specified Input

Finally, we applied XAI methods to inspect individual images from the TEST1 group. For this purpose, we chose fold six, as it demonstrated both high accuracy and stability. From the TEST1 group, we selected 2 representative samples, one per class, which entered the inspection of the prediction process using the occlusion method.

**Figure 3.**
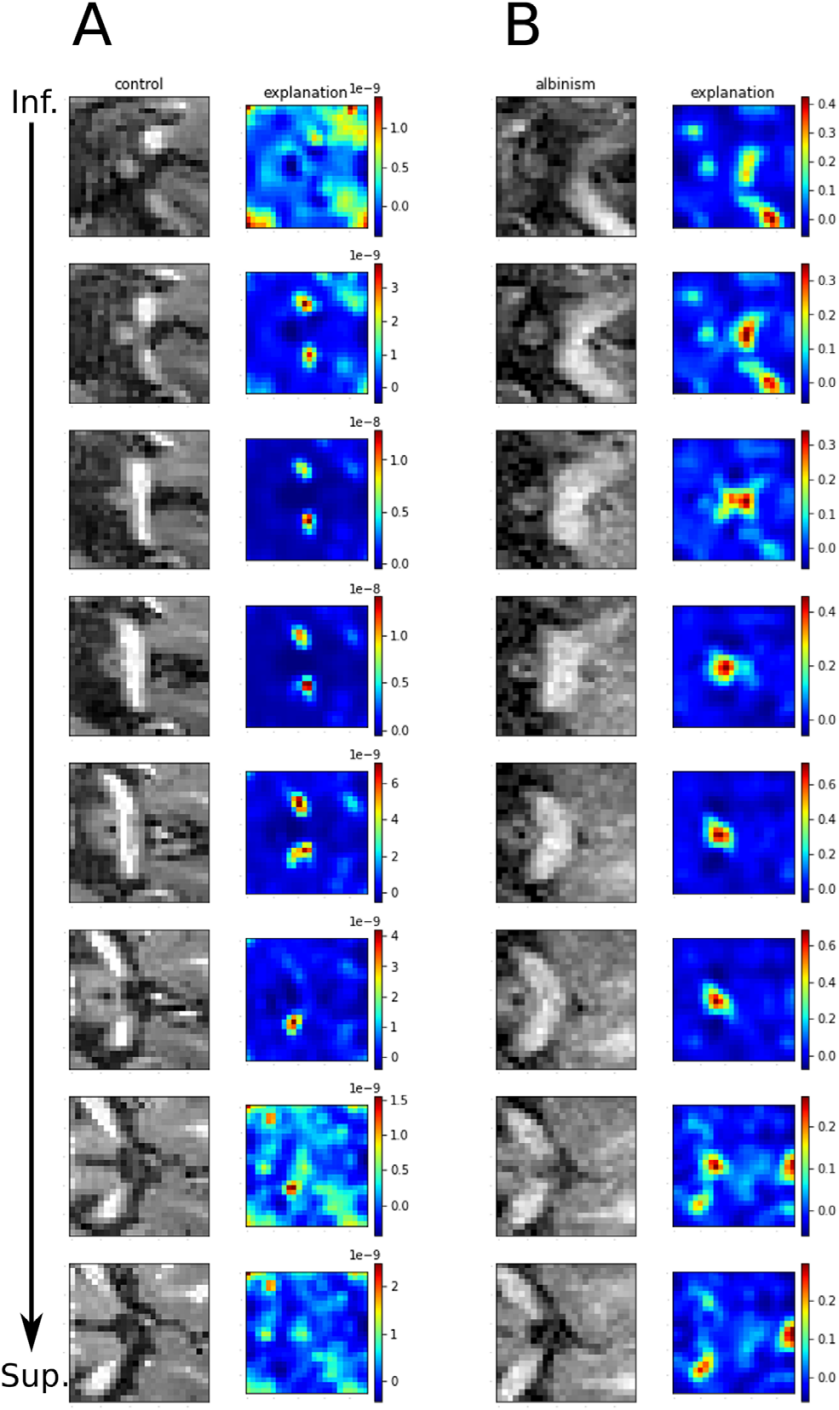
Explanation of predictions for selected control and PWA input with occlusion method (fold 6, TEST1). (**A**) Sample control image. (**B**) Sample albinism image. Each panel in the left column demonstrates subsequent slices of T1w image, from inferior to superior. The right hand-column presents the attributions map obtained with occlusion approach. Warmer colors mark areas that mostly contribute to predictions. The predictions are driven by the areas containing the white matter which correspond to the optic chiasm and optic tracts.

The results are depicted in Figure 3, where the most relevant areas are false color-coded with warm colors, that is, maximally red. In the case of the controls, the regions driving the prediction of “control” are the superior (dorsal) parts of the optic chiasm body and the optic tracts. For albinism, the most relevant region for the prediction of “albinism” is the body of the optic chiasm, although the attributions map also highlights optic nerves and tracts.

## Discussion

We have demonstrated the applicability of CNNs for the direct detection of chiasmal malformations from anatomic MRI scans in PWA. Here, we will be discussing three essential questions for the tested methods: (i) comparison of performance of the proposed method to alternative diagnostic procedures, (ii) reliability of the predictions of the proposed CNN (CHIASM-Net), and (iii) applicability of the method in modern clinical environments. A supplementary discussion of specific study results is provided in “Supplement 8: Detailed discussion of CHIASM-Net's performance.”

### Comparison to Alternative Procedures to Detect Optic Chiasm Abnormalities

An important aspect of the evaluation of each method is its comparison to other approaches. In this case, it implies the exploration of alternative methods of detecting chiasmal malformations from brain images in the absence of functional testing. At present, the only method applied for this purpose involves highly time-consuming diffusion MRI measurements, which requires specific MRI and computational methods. In a pioneering study, Ather et al.^13^ reported an accuracy of detection of albinism at 75%, which, as concluded by the authors, was below the necessary minimum for clinical application. This approach was revisited by Puzniak et al.,^14^ who also reported an area under the characteristic curve (AUC) of 75%. The results were, however, encumbered by the small sample size (a common problem in the research of rare congenital diseases, such as albinism). Notably, due to limited sample size, the provided estimates of accuracy were obtained for the same data on which the model was trained, and as such does not reveal the true performance of the method.

Unlike diffusion MRI data, anatomic MRI data (T1w anatomic images) are more readily acquirable and available in a clinical environment. Very few studies have so far made use of anatomical features for a quantitative comparison of the optic chiasm in PWA and controls.^16^^–^^19^ Applying standard techniques, group differences are reported, but did not assess individualized detection of albinism for diagnostic purposes. In contrast, a recent study employing a DL-based approach^15^ provided proof-of-concept for such an approach which motivated the present study. Specifically, it was demonstrated that U-Net CNN trained on control data for segmentation of optic chiasm performs in a different manner for PWA and healthy controls. This was demonstrated by errors in segmentation of the optic chiasm in PWA, which were not evident for controls. The separability of data points representing correctness of segmentation was measured by fitting and evaluating performance of a C-support vector machine on the same set of data. Importantly, although this step used machine learning methodology, its only purpose was to quantify the separability. The reported metrics with accuracy of 0.84, precision of 0.82, recall of 0.9, and specificity of 0.78^15^ indicate that, although majority of data points can be well grouped, there is a portion of borderline cases which might be assigned to the wrong group. Whereas we are not able to falsify/verify that hypothesis, we note that the classification error reported in the present study is in a similar range to the one described in the above study. Until further research is performed, we can only speculate whether it might be the variability of chiasma malformations that limits the classification.

In summary, we note that the CHIASM-Net's performance in detecting chiasmal malformations surpasses the previous alternative approaches from anatomic images, that is, analyzing T1-anatomy-based chiasm geometry^16^^,^^18^ and diffusion-weighted MRI of the chiasm.^13^^,^^14^ Given that a larger albinism sample size will further improve CHIASM-Net's performance, we conclude that the CHIASM-Net-based approach of the present study is of promise for future applications.

### Reliability of the Predictions

The applicability of a deep neural network in a clinical environment is determined not only by an acceptable quantitative performance. Critically, it is also determined by the evidence of the involvement of physiologically meaningful input features behind the prediction outcome. In the case of detecting chiasmal malformations, the relevant input features would be limited to white matter voxels of the optic chiasm.^16^ Inclusion of voxels outside the chiasm, for example, blood vessels or surrounding tissue, would indicate usage of unreliable features and, consequently, non-trustworthy predictions. Specifically for the chiasm, Schmitz et al.^16^ reported that the albinotic chiasm is thinner and that the angles between the optic tracts are wider. To verify whether these are the characteristics of the relevant input provided for training and evaluation, we averaged input images and calculated their difference. Interestingly, the visualization of residuals showed close resemblance to the images from the previous report Schmitz et al.^16^ Specifically, we observed higher white matter density in the lateral parts of the optic tracts in controls, whereas in albinism the medial parts were more expressed. This is in accordance with the previously reported changes in the angles of optic tracts reported previously. Additionally, we observed higher intensity of the voxels in the body of optic chiasm, indicative of higher density due to its smaller diameter and an increased crossing. We take this specificity of our findings as an indication that CHIASM-Net's predictions are physiologically plausible, as they are dominated by the previously reported anatomical optic chiasm deviations between albinism and controls.

### Clinical Applicability

Our CNN-based method to detect optic nerve misrouting in albinism with T1w anatomic MRI scans carry several advantages compared to other MRI-based approaches, as in dMRI or functional MRI (fMRI). These benefits include: (1) shorter acquisition times (approximately 10 minutes) with standard protocols, (2) easier applicability in patients with visual function deficits, (3) data availability, as the method can be applied on T1w images acquired for a different primary reason, and (4) fully automated computations with faster predictions and very little involvement of human factor except for final assessment and verification of the results. In addition to these factors, the findings from our study emphasize the ever-increasing feasibility and practicability of CNN-based methods for clinical diagnostics.

### Limitations

A large quantity of qualitative data is critical for the development and performance of data-driven DL-based methods. This requirement is a particular challenge in rare diseases, such as albinism. In order to deal with this issue of data scarcity, we combined data from our past work^27^ with those from an independent study,^13^ to obtain the biggest MRI dataset on albinism used in the so-far reported studies. Despite this achievement, we note that MRI volumes from 32 PWA is insufficient for thorough training and testing of CHIASM-Net in order to detect chiasmal malformations. Although this limitation was to some extent mitigated by our experimental design (e.g. performing training on widely accessible MRI scans of controls and subsequently fine-tuning the created network with smaller sample), we note that ultimately this problem can be resolved only by the acquisition of more MRI data on albinism and ideally sharing it with the wider scientific community. Additionally, when training neural networks, in particular in conjunction with the low size of input patches, it is necessary to consider overfitting as a potential confound. To mitigate this risk, we have used several steps, at the level of both architecture and training design. Finally, we have confirmed the lack of overfitting by analyzing the recorded learning curves. A detailed discussion on specific steps and analysis of learning curves is provided in the supplementary materials (Supplement 9: Overfitting).

## Conclusions

In this study, we developed a novel AI tool, CHIASM-Net, to detect chiasmal abnormalities from T1w MRI scans and evaluated its performance with a classifier metric. The neural network relied on physiologically plausible input features in the decision making process and resulted in a high performance in classifying normal and albinotic chiasms. The present study is intended to introduce an AI-based tool for albinism diagnostics from anatomic images and to motivate further applications in optic chiasm diagnostics. Notably, this approach for albinism detection might also be retrospectively applied to brain anatomic T1w MR images acquired in the past for different purposes.

## Supplementary Material

Supplement 1
