# Supplementary material for: CHIASM-Net: Artificial Intelligence-Based Direct Identification of Chiasmal Abnormalities in Albinism
Source: Invest Ophthalmol Vis Sci. 2023 Oct 10;64(13):14. doi: 10.1167/iovs.64.13.14 (PMC10573586; doi:10.1167/iovs.64.13.14)
Supplement: Supplement 1 [file iovs-64-13-14_s001.pdf]

Supplement 1: Data sources

Although both Computed Tomography and MRI are capable of providing anatomical images of the chiasm, the former has limited contrast for chiasm imaging [34]. This restricts the applicability of CT-based AI tools for optic chiasm segmentation [35,36]. Consequently, we based our study on T1-weighted (T1w) MRI images, which are typically acquired for the whole brain volume, and were gathered for both controls and PWA. Following the approach of McClure et al. [37], data of controls was acquired from multiple, openly available sources. Importantly, since the controls were required to only show the typical organization of the visual system and optic chiasm, the datasets used included neuro-divergent participants, such as people with autism spectrum disorder [21]. The datasets on albinism, due to its notable rarity (approximately in range of 1:14 000 or 1:20 000 [4,29]), were limited to a publicly available CHIASM dataset [27] and the non-public data used in a published study on albinism [13]. Information on the data sources is detailed in **Suppl. Table 1**.

Preprocessing steps of the T1w images are detailed in the **Supplement 2: Preprocessing of T1w MRI images.** In brief, all the images were resampled to a common resolution of 1.0 mm isotropic [38] and skull stripped [39,40], followed by an automated localization of the optic chiasm [15,41]. The information on the location of optic chiasm was used for the creation of (a) binarized masks of brain with removed optic chiasm region and (b) patches of the T1w images that comprised the optic chiasm only. These were used for the training of the CNN's feature extraction and classification modules, respectively (see **Supplement 3: Feature extraction module** and **Supplement 4: Classification module**).

Dataset	Source	Number of controls	Number of PWA
ABIDE [21]	COINS Data Exchange [42,43]	355	-
Ather et al. [13]	-	20	23
CHIASM [27]	brainlife.io [44]	8	9
COBRE [22]	COINS Data Exchange [42,43]	60	-
HCP [23]	brainlife.io [44]	1065	-
Collegiate athlete brain data [24] (referred to as "Athletes")	brainlife.io [44]	42	-
Mind-Brain-Body dataset [25]	brainlife.io [44]	133	-
MCIC [26]	COINS Data Exchange [42,43]	25	-
	Total	1708	32

Suppl. Table 1. List of the used datasets, the download source, and the number of participants from each group.

All images were obtained from publicly available datasets, except for the data from our previous study [13].

Supplement 2: Preprocessing of T1w MRI images

To account for the cross-dataset differences and improve the training process, the data were subjected to preprocessing. Initially, all acquired T1w images were resampled to 1.0 mm isotropic voxel using *mrgrid* tool (MRtrix software [38]). The resulting images were processed using a bet2 tool with a threshold of 0.15 (FMRIB's FSL software [39,40]) to create skull-stripped brain images and corresponding binary brain masks.
The next step of preprocessing involved the localization of the optic chiasm in the T1w images, which was achieved in one of several ways, depending on the characteristics of dataset:

- Manual: For data from CHIASM [27] and [13] datasets, we used the results of manual segmentation of the chiasm for its localization in the brain. Specifically, for the CHIASM data we used the existing manual mask of the chiasm provided in [15], whereas for data from [13] the manual segmentation was performed by a trained operator according to guidelines outlined in [15].
- Automated segmentation with FreeSurfer: For the data from the HCP dataset, the masked images of optic chiasm were obtained through FreeSurfer's segmentation [41] of the original whole-brain MRI images and were provided on brainlifeo.io together with the original data. Theoretically, FreeSurfer segmentation could be as well applied to the rest of the brain MRI datasets. Practically, however, with an average of 8 hours per segmentation of a single subject, performing this for all the remaining datasets would take approximately 225 days, which prevented us from following up with this method.
- Segmentation with CNN from Puzniak et al. (2021)[15]: In order to efficiently locate chiasm in the remaining T1w images, we used a CNN developed for the purpose of chiasm segmentation [15], which performed the desired operation in seconds. However, as the applied CNN was primarily designed for segmentation of HCP data only, and was not validated on other datasets, this was an experimental approach. Interestingly, the assessment of the results revealed robust results for most of the remaining datasets, e.g. Athletes, whereas for some datasets, such as COBRE, the CNN was consistently failing for all subjects.
- "Limited" manual: Due to the experimental nature of segmentation with CNN described in the previous item, all chiasm segmentation results were manually validated. In case of erroneous or missing segmentation results, the segmentation was done manually by selecting a single voxel in the centre of the chiasm, which was sufficient for the localization of the chiasm in the image.

Effectively, the procedures described above led for all brain images provided binary masks including the location of the optic chiasm in the brain. This information was used for the next preprocessing step, which involved the extraction of patches of T1w images containing chiasm. Specifically:

- For each masked patch, we determined its centre (centroid of the bounding box).
- From each T1w image we extracted a patch of size 32x32x12 (32 x 32 x 12 mm) that was centred on the previously determined central point of the mask. The size of patch was deliberately bigger than the size of the designed input to CNN (24 x 24 x 8 mm) - this was to ensure that image transformations done as part of data augmentation will not introduce empty voxels to the input.

Notably, the patch size was not optimized in the analysis. The ultimate dimensions of 24 x 24 x 8 mm were chosen to ensure that the patch can fully cover the optic chiasm (reported average width and height equal to 15.0 and 3.5 mm, respectively [28]), as well as adjacent parts of optic nerves and tracts. While fixing the height of the patch, we also considered that the optic chiasm's plane is not parallel with the axial plane of MRI images' native space. The average of the input patches (**Figure 1**) and the samples of selected individuals (**Figure 3**) demonstrate that the selected the dimensions indeed fully covered the optic chiasm and adjacent parts of optic nerves and tracts.

Finally, we ensured that the dimensions are divisible by 4 which slightly optimized the performance of network (since operation of max pooling with kernel of 2 x 2 x 2 did not require padding) and more importantly established a clear link between the latent representation (6 x 6 x 2), receptive fields (4 x 4 x 4) and input image dimensions (24 x 24 x 8).

In the final step, we modified each of the previously obtained binary brain masks by marking a spherical region of radius 23.4 mm (half of diagonal of 32 x 32 x 12 mm patch) centred on the previously determined central point of the chiasmal mask. This modified binary brain mask was used to guide the sampling of patches from the brain volume, which was done for the purpose of feature extraction.

Importantly, the above outline describes only the general preprocessing steps applied to all data before the training. Each specific training run used an additional custom data preparation procedure, which are described in corresponding sections.

Supplement 3: Feature extraction module

Architecture

The feature extraction layers were modelled after the 3D U-Net [45,46], a well-established architecture for medical imaging [47], which we have previously demonstrated to be sensitive to structural chiasmal abnormalities typical for albinism [15]. The default architecture of 3D U-Net was modified to accommodate the low size of the input by:

- Reduction of its depth: We tested two variants with respectively two and three layers, as opposed to the original four. The optimal number of convolutional layers for our purpose was determined to be two.
- Removal of the residual connections: The purpose of using residual connections in the U-Net [45] was to preserve high resolution features of input. However, this is not necessary for the input of size 24x24x8 and was therefore removed.
- Replacing max pooling with average pooling: While the original architecture used max pooling layers [48], we decided to replace them with average pooling layers in order to be able to apply a wider range of Explainable AI (XAI) techniques.
- Reduction of the number of feature maps: Given the considerably lower input dimensions than in original 3D U-Net, we accordingly reduced the number of feature maps created within the CNN. Specifically, we tested two variants: (a) 4 feature maps created in the first layer and passed to the second and (b) creation of 2 feature maps in the first layer and doubling their number in the second. To distinguish between two variants, we introduced the naming convention that is formatted as <number of feature maps in the first layer>_<multiplier of number of maps between first and second layer>. Accordingly (a) would correspond to “4_1” while (b) would be described as “2_2”. Importantly, the size of latent representation for both variants is identical (6 x 6 x 2 x 4 feature maps = 288 voxels) which corresponds to 1/16 of the number of voxels in the initial image.

The details of the autoencoder’s architecture are provided in **Supplementary table 2**.

Stream	Functions		Kernel size	Stride	Padding
Encoding (analysis)	U-Net block	3D convolution	3	1	1
		3D batch normalisation	-	-	-
		ReLU	-	-	-
		3D convolution	3	1	1

		3D batch normalisation	-	-	-
		ReLU	-	-	-
	Pooling	Average pooling	2	2	0
	U-Net block	3D convolution	3	1	1
		3D batch normalisation	-	-	-
		ReLU	-	-	-
		3D convolution	3	1	1
		3D batch normalisation	-	-	-
		ReLU	-	-	-
Decoding (synthesis)	Upconvolution	3D transposed convolution	2	1	2
	U-Net block	3D convolution	3	1	1
		3D batch normalisation	-	-	-
		ReLU	-	-	-
		3D convolution	3	1	1
		3D batch normalisation	-	-	-
		ReLU	-	-	-
	Convolution	3D convolution	1	1	0

Suppl. Table 2. Detailed architecture of autoencoder.

Training

Feature extraction training was performed in an unsupervised fashion. In this process, we trained the custom autoencoder architecture to encode the input image (here: a 24x24x8 patch of MRI image) to a

latent representation (dimensions of 6 x 6 x 2 x 4) and decode it back to the original image. Ultimately, for the feature extraction module we only used parts of the network that represent encoding stream (and compress input image to latent representation), while the decoding stream was discarded.

Intuitively, the autoencoder should be trained only on the optic chiasm images. This approach, however, severely reduces the available data to 1740 (1 per participant) patches only, which can possibly limit the effectiveness of training and cause further problems, such as overfitting. It should also be noted that the task of image encoding does not necessarily have to be trained on optic chiasm images only, as the underlying principles of MRI imaging apply to all other brain structures. Consequently, the problem of feature extraction can be generalized for all the brain regions, which can as well be used for the training. This approach provides a valuable advantage of using complete information from the acquired 1740 whole-brain images in training and would result in a theoretically more generalizable and robust network than the one trained on optic chiasm images only. Accordingly, we trained the autoencoder on patches from the whole brain volume, but with two important modifications.

Firstly, since the selected training approach was not specific to optic chiasm anymore, we excluded this structure from the training data. This step enabled us to assess the autoencoder’s performance on unseen brain structures (optic chiasm images) without the danger of data leakage. Secondly, the introduced separation of data used for feature training (brain without chiasm) from data used for later training of classification (chiasms only) allowed us to use different data splits for both training phases. This provided an advantage of tuning the definitions of groups and their sizes to specifics of each training phase (e.g. feature encoding trained on more samples or classification trained on different test-train splits).

Technically, the MRI patches for training the feature extraction module (autoencoder) were generated by uniform sampling of voxels across the whole brain volume (skull-stripped). Each selected voxel served as the centre of 24 x 24 x 8 patch, which was subsequently used as a single training sample. The exclusion of the optic chiasm was achieved by drawing a sphere of radius of 23.4 mm (half the diagonal of the patch) and the probability of sampling was set to 0 in this zone. This ensured that the complete images of optic chiasm could not be captured by patches of size 24 x 24 x 8, and consequently were excluded from the autoencoder training. The simpler alternative to this approach would involve direct masking of the region of optic chiasm (e.g. by volumetric mask of 0's), and unrestricted sampling of the whole-brain volume. This would, however, introduce false information to the training data, with a potential to impair the training. For this reason, we decided to adjust the sampling strategy rather than modify the input images.

For training and testing, we used the following TRAIN / DEV-TRAIN / DEV-TEST / TEST data splits. 80% of data from datasets not containing PWA (ABIDE, Athletes, HCP, COBRE, Leipzig, MCIC) was used for training (TRAIN), 10% of it was included in the DEV-TRAIN group for monitoring of training performance and early stop criterion, and the remaining 10% were included in the DEV-TEST group to assess the performance of the trained autoencoder on the unseen data from seen datasets. As for the datasets containing PWA, the data was used only for testing of the network’s performance. Specifically, 15% of it was included in the DEV-TEST group to repeatedly evaluate the performance on unseen sample from

unseen dataset. The remaining 85% was kept away from the training phase (TEST) and was used in a one-time test of autoencoders to obtain a realistic assessment of the network's performance on the unseen data from unseen source.

Prior to training, all images underwent data preprocessing, which involved histogram standardization [49] to mitigate any cross-site dataset differences and rescaling of voxels intensity (range: [0;1]) for stability of training. Additionally, the data from TRAIN group underwent data augmentation [50], which involved random flipping along any axis (applied in 50% of cases, if applied each axis was flipped with 25% probability). Finally, the 24x24x8 patches from the whole-brain volume (excluding the ones which would include complete optic chiasm) were sampled using "WeightedSampler" from the torchio package [51] .

To monitor the loss between the input and reconstructed image during training we used Mean Square Error (MSE). The weights were updated with the Adam optimizer [52] with a fixed learning rate step of 0.001. The maximal training length was set to 50 epochs, however we also used early stopping criteria to cease training if the MSE on the dev-train data could not be reduced for 5 consecutive epochs. The implementation was in pytorch 1.10.0 [53].The complete training was performed twice, for two different sets of parameters defining the custom CNN architecture we employed, specifically variants “4_1” and “2_2”. The performance of the trained autoencoder was evaluated on randomly sampled images from the held-out TEST group. Additionally, we evaluated the performance of the autoencoder on the specifically prepared chiasm images from the held-out TEST set. Importantly, after this explorative step any further changes to feature extraction module were not allowed, as it would incur data spoiling. The quality was measured by the MSE (see **Evaluation metrics**), while the detailed results are described in the **Results** section.

Supplement 4: Classification module

The final step involved the design and training of the classification module which takes the extracted features of the chiasms as an input and outputs a classification score. For this purpose, we reduced the previously developed autoencoder module to the analysis (encoding) stream only and, with its weights frozen, connected through a flattening operation to design a classification module composed of fully-connected nodes. The details on the architecture and training are provided in the subsequent sections.

Architecture

The classification module comprised of fully-connected nodes and was tested in two variants: (a) without hidden layer, (b) with a single hidden layer. In case of (a) all elements of the one-dimensional vector resulting from flattening the latent representation of the autoencoder were fully-connected to a single node outputting a classification score. For (b) the one-dimensional vector was fully connected to nodes of hidden layer (of arbitrary number N, we tested N = 16 and 256), where, after summation and application of rectified linear unit (ReLu) activation function [54,55], the nodes of the hidden layers are connected to a single output neuron. Analysis of different variants and combinations of hyperparameters allowed us to not only fine-tune the network, but also gain a better understanding of the optimal values of the parameters.

Training

The next training step involved updating weights of the newly added classification layer (note that the weights of feature extraction module were frozen at this stage). This training was done using only patches of T1w images fully covering the optic chiasm (size 32x32x12), prepared as described in the **Data** section. Additionally, since images of optic chiasm were strictly excluded from feature extraction training (except one time for final testing) we were able to use new data splits without the danger of data spoiling. We used this opportunity to create new, optimized data splits, which supported balancing of classes in training and testing data sets (32 PWA vs 1708 controls).

Specifically, we created two groups for training: (a) TRAIN group, (b) DEV_TRAIN group, and two groups for evaluation in two distinct scenarios: (c) TEST1 and (d) TEST2.

a) TRAIN data was used exclusively for training of weights of the classification layer. Importantly, due to a high-class imbalance, the samples from the minority dataset (PWA) in TRAIN data were upsampled by a matching factor (approx. 55) to balance the two existing classes, which is beneficial for stable training.

b) DEV_TRAIN data was used to monitor the performance of the network over training and ultimately select the best set of weights for the classification layer. Similar to the TRAIN data, the DEV_TRAIN data was upsampled by an appropriate factor (approx. 54) to prevent bias introduced by a class imbalance.

c) TEST1 consisted of 4 samples of PWA and 4 controls (with 3 samples in each group coming from Ather et al. dataset, and remaining one from the CHIASM dataset). This allowed for the

evaluation of network's performance on a balanced set of data excluded from training, though acquired from the same source as the training data.

d) TEST2 sample emulated real life scenario. While we were not able to simulate the true proportions of (approximately between 1:14000 and 1:20000 [4,29]), we used a single PWA sample from CHIASM dataset and remaining samples (not involved in training) from all control datasets to obtain a proportion of 1:214.

The training of the classification module differed from that of the feature extraction module in three important aspects. Firstly, the two datasets with PWA (Ather and CHIASM) were not used separately for testing, but were mixed and used for both training and testing. Effectively, we could not investigate the performance of the network on the relevant data obtained from a source other than the training data. However, this approach allowed us to increase the number of training samples, which we deemed more important at this stage of exploring the general application of CNNs to albinism diagnostics.

Secondly, to obtain more robust estimates of CNN's performance, we used an 8-fold validation approach. Specifically, the samples (both controls and PWA) from each dataset were divided into 8 equal subsets (except for the CHIASM, which was divided in 9). Out of those:

- 6 subsets from each dataset were included in the TRAIN group
- 1 subset from each dataset was included in the DEV_TRAIN group
- TEST1 group included the remaining 1 subset from Ather et al. and 1 out of 2 remaining subsets for CHIASM
- TEST2 group included the remaining subset of CHIASM and remaining single subset from all other datasets

Once the groups were created, we performed the training and evaluated the CNN's performance using defined TEST1 and TEST2 groups. This whole procedure was repeated 8 times, each time assigning different samples to defined groups. By averaging the evaluation metrics from the repetitions, we obtained more robust performance estimates. The exemplary data splits for a single run of k-fold training is presented in **Suppl. table 3.**

Thirdly, to mitigate the class imbalance in the TRAIN and DEV_TRAIN datasets, the data of minority class (PWA) was upsampled to make their number equal to the datasets of controls. This allowed for the reduction of the impact of class imbalance on the training. It should be noted that this step was not applied for neither TEST1 (since the datasets selected for this group were already balanced) nor TEST2 (since this group was designed to mimic the class imbalance observed in real world) groups.

	TRAIN		DEV_TRAIN		TEST1		TEST2	
	CON	ALB	CON	ALB	CON	ALB	CON	ALB
ABIDE	265	-	45	-	0	-	45	-
Ather et al.	14	17 (X*)	3	3 (X*)	3	3	0	0
CHIASM	6	6 (X*)	1	1 (X*)	1	1	0	1
COBRE	44	-	8	-	0	-	8	-
HCP	798	-	133	-	0	-	134	-
Collegiate athlete brain data	30	-	6	-	0	-	6	-
Leipzig	99	-	17	-	0	-	17	-
MCIC	18	-	3	-	0	-	4	-
	**1274**	**23 (X*)**	**216**	**4 (X*)**	**4**	**4**	**214**	**1**

Suppl. Table 3. Exemplary split of data for single run of 8-fold validation. Different runs made use of different data-sets for given groups.

* PWA datasets were upsampled by a dynamic factor to match the number of used control data.

Before training, all samples were subjected to data preprocessing and augmentation. The preprocessing involved:

- transformation of all samples to a common canonical (RAS+) orientation (done with torchio package [51])
- standardization of intensities of all samples [49]. This was achieved by training the standardizer on all samples, and subsequent transformation of all samples with obtained standardizer. Accordingly, this operation allowed us to mitigate cross-site differences.
- rescaling of voxel intensities to range [0;1]

Additionally, the samples from the TRAIN and DEV_TRAIN subsets were subjected to data augmentation, which involved combination of a random: (a) translation in any direction up to 2 voxels, (b) rotation up to 5 degrees in any position, with the axis of rotation passing through the middle point of a patch image.
At the end of preprocessing/data augmentation procedure, all samples were cropped from their original shape of 32x32x12 to the one used previously in feature extraction training: 24x24x8. As indicated before, the initial excessive size of input patches allowed for the application of random transformations to samples without the risk of introducing empty spaces in the final sample.

The training used an Adam [52] optimizer and was performed multiple times with various learning rate parameters of [0.001, 0.0001, 0.000001 and 0.00001]. Importantly, only the weights of the newly appended classification layer were updated during training. For loss function, we used binary cross entropy (BCE) [56], which is an adequate measure for assessing a score against a binary output (0 for control and 1 for albinism). The training length was set to a maximum of 8000 epochs, with the early stopping criterion of 1000 epochs. If no improvement in the performance was made for 1000 consecutive epochs the training was stopped. The performance during the training was monitored on the DEV_TRAIN sample. Upon training completion, the weights corresponding to the best score on this sample were selected (see **Results**).

Supplement 5: Evaluation of feature extraction module

The purpose of the feature extraction module was to achieve an efficient encoding of the original image to a compressed latent representation, thereby enabling a possible lossless reconstruction of the original image. The quality of the described procedure of autoencoding was assessed by the comparison of the original and reconstructed images. For this purpose, we used Mean Squared Error (MSE) loss function, as previously used in the module training, which for original image $I(x, y, z)$ and the reconstructed image $\hat{I}(x, y, z)$ is defined as:

$$MSE(\hat{I}, I) = \frac{1}{I \cdot J \cdot K} \sum_{i=0}^{I} \sum_{j=0}^{J} \sum_{k=0}^{K} \left[\hat{I}(i, j, k) - I(i, j, k)\right]^2$$

(Suppl. Eq. 1)

Where $\hat{I}(x, y, z)$ and $I(x, y, z)$ describe the intensity value of voxel with coordinates $I(x, y, z)$ in images $\hat{I}$ and I, respectively. While the absolute values of MSE are not directly interpretable, they allow for direct comparisons of different approaches and identification of the best one (i.e., one with the lowest MSE). To compensate for this limited interpretability of MSE, we additionally calculated and visualized the residuals between the original and reconstructed images and subsequently used it for visual inspection. In an ideal scenario, the decoded image would capture all relevant features of the input image, except for noise. Effectively, the residual map for ideal encoding would consist only of noise. In case of less-than-ideal performance, the residual map would contain shapes/contours of respective brain structures present in the original image, up to the point where the residual map is identical with the original image (meaning that the decoded image is empty).

Supplement 6: Results for feature extraction module

The performance of the trained autoencoders with different combination of hyperparameters was assessed on two separate groups:

- DEV-TEST comprising images of both controls and PWA that were excluded from the training.
- TEST comprising images of both controls and PWA from Ather et al. and CHIASM datasets. It should be noted that none of the images from those two datasets was included in training, as such images from the TEST dataset were not only unseen, but were also coming from the previously unseen dataset source.

The best performing autoencoder was selected and adapted as the feature extraction module. A quantitative (average MSE per image) overview of assessment results of the tested autoencoders is provided in the **Suppl. Figure 1A**, with the detailed quantitative results being listed in the **Suppl. Table 4**.

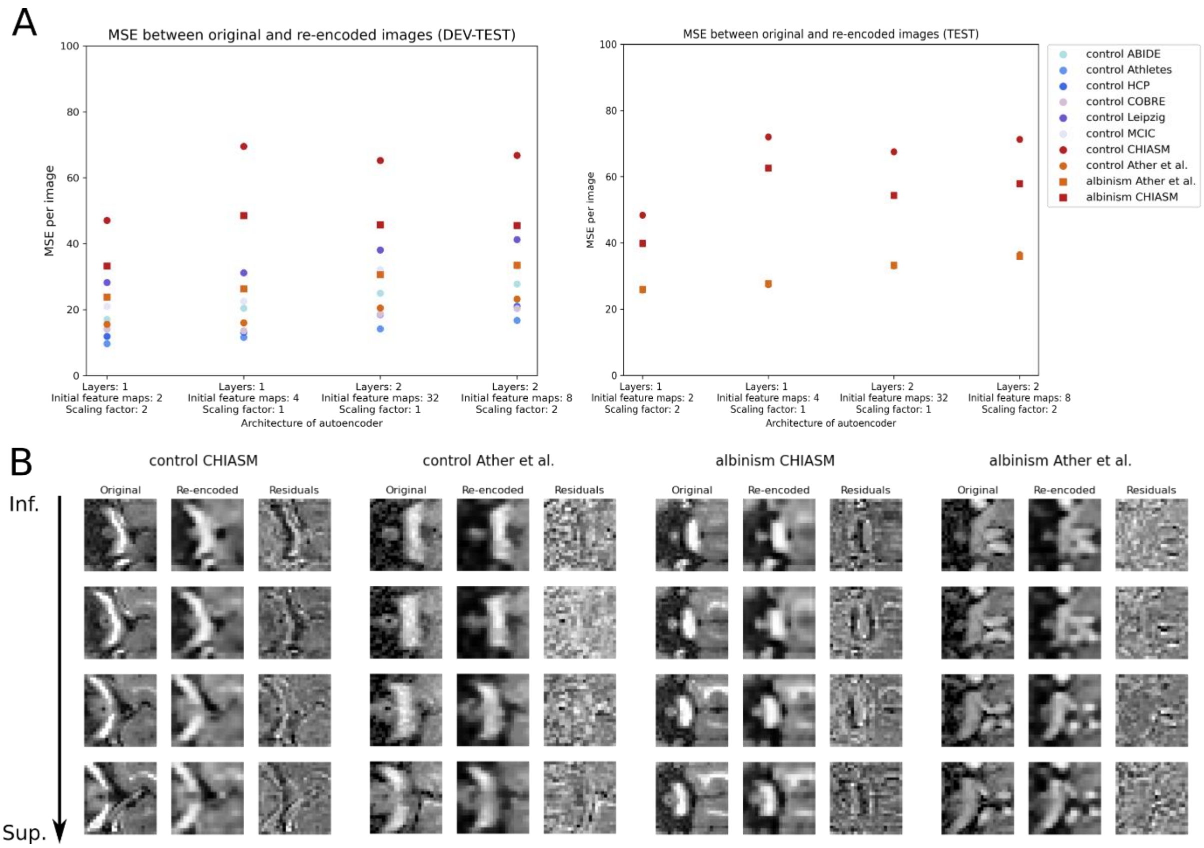

Suppl. Fig. 1. Qualitative and quantitative overview of performance of the tested autoencoders.

(A) Plots of average MSE per image between original and re-encoded images. The y-axis displays the mean MSE between the input MRI patch capturing optic chiasm and re-encoded patch, the x-axis corresponds to tested autoencoders with different hyperparameters combinations. The left-side plot corresponds to results obtained for images from DEV-TEST group, the right-side plot displays the results for images from the TEST group (notably, the samples from datasets CHIASM and Ather et al. were explicitly excluded from training phase and were used only for evaluation). **(B) Qualitative comparison of original and re-encoded images for samples from TEST data encoded with Encoder with the hyperparameters (Encoder - 1 Layer; Initial Feature Maps – 2; Scaling Factor - 2).** The plots are shown in 4 groups displaying, from left to right, sample of control data from CHIASM and Ather et al. datasets, respectively, and sample of albinism data from CHIASM and Ather et al., respectively. Each group is divided in 3 columns, with left-most column showing the original patch of MRI image, middle one showing the re-encoded image (an output of the autoencoder) and left-most showing residuals (difference

between original and re-encoded image calculated by subtraction). The top row displays most inferiors, with subsequent rows displaying gradually superior slices.

Out of 4 tested autoencoder architectures the best performance (indicated by the lowest average MSE for all distinct subsamples from test) was observed for the encoder with the following hyperparameters: Encoder - 1 Layer; Initial Feature Maps – 2; Scaling Factor – 2 (**Suppl. Figure 1A** and **Suppl. Table 4**). This observation was further validated by visual inspection of the residual maps, which, particularly for images from Ather et al., demonstrated high quality encoding of the original images (**Suppl. Figure 1B**). Specifically, the residual images are filled mostly with noise and show little to no sign of brain structures, which indicates good reconstruction. Accordingly, we decided to use decoding stream from “Encoder - 1 Layer; Initial Feature Maps – 2; Scaling Factor 2” as a feature extraction module in the designed CNN for classification of chiasmal malformations.

Supplement 7: Results for classification module

As stated in methods, the selection of the optimal hyperparameters involved testing a combination of different architectures and learning rates. The tested combination was indicated in the name of network as *<number of layers in feature encoding module>_<multiplier of feature maps in feature encoding module>_<number of hidden layers>_<number of nodes in hidden layer>_<learning rate>.* For instance, „1_2_2_1_256_1e-05" represents network with 1 layer in encoding module that initally creates 2 feature maps, doubles this number by a factor 2 in the latent representation and is fully connected to the classification module with 1 hidden layer with 256 nodes that was trained with a learning rate of 1e-05 . **Suppl. Figure 2** presents the accuracy, precision and recall-score obtained from the trained classifying modules (see **Methods** for details on the development of the classification module). The illustrated results were obtained for the TEST1 group for 1/8 runs performed during the k-fold validation.

A

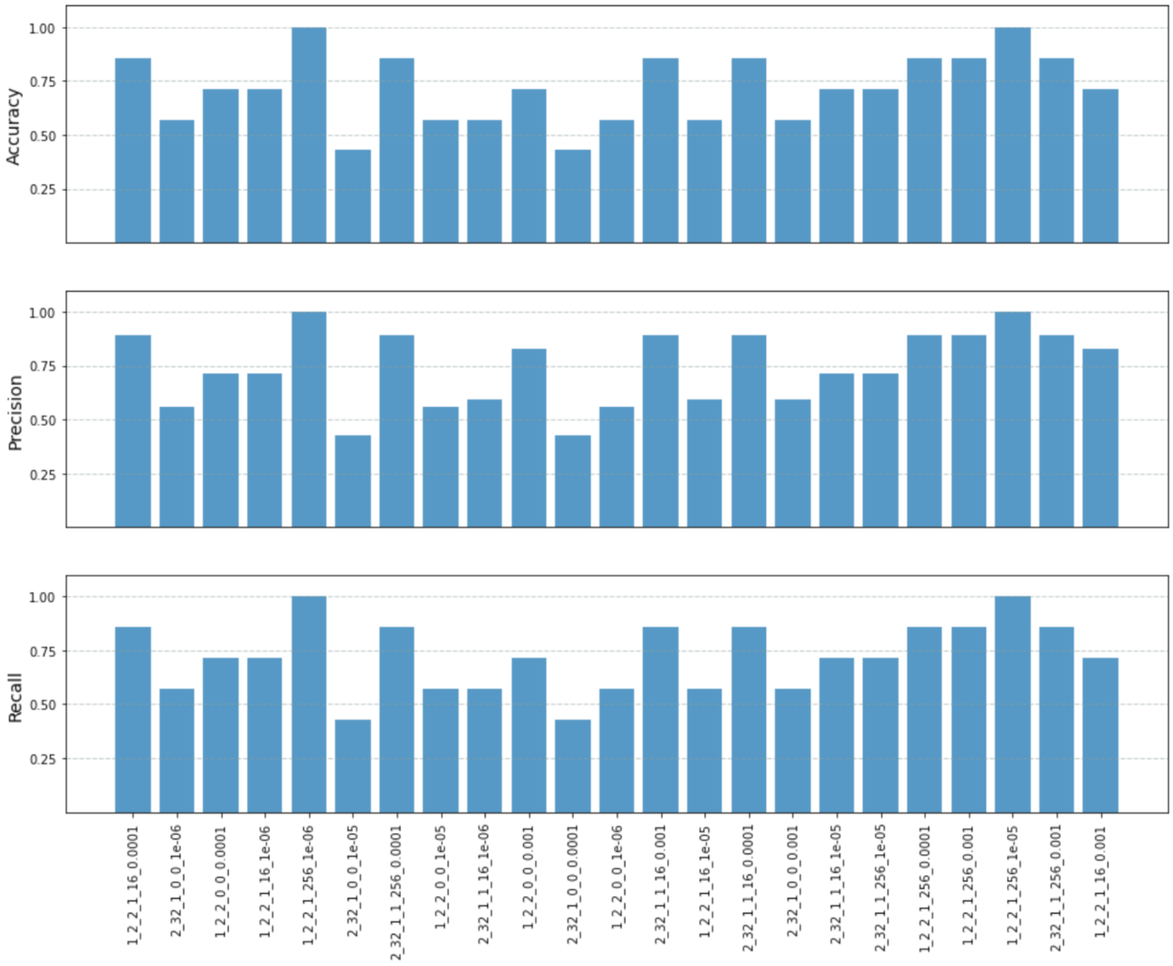

Suppl. Fig. 2. Evaluation metrics, training loss and explanation of predictions for selection of optimal classification module. Accuracy (top row), precision (middle row) and recall (bottom row) for multiple tested networks (x-axis).

Based on our results, classifying fully-connected networks with a hidden layer performed better than those without one, with best results obtained for 256 nodes in hidden layer for learning rates of 1e-06 and 1e-05. Learning rate of 1e-06 as compared to 1e-05 extended the duration of training by a factor of 5, but at the same time did not provide substantial improvements in convergence or end accuracy. Effectively, we determined the optimal architecture to consist of encoding module of 1_2_2_1 and a classifying layer with 256 hidden neurons and a single output neuron, trained with Adam optimizer with learning rate of 1e-05.

Supplement 8: Detailed discussion of CHIASM-Net’s performance

The evaluation of the trained CNN with 8-fold validation, using class-balanced TEST1 group yielded an accuracy and F1-score of 85 ± 14 %, precision of 90 ± 14 % and recall of 81 ± 18 % (Mean ± Standard Deviation; **Table 1)**. The findings suggest that the method tested has potential for future applications in detection of chiasmal malformations. Our positive opinion is additionally supported by the fact that the reported values are an average of 6 well-performing folds and 2 folds having considerably lower metrics (folds 1 and 5 with AUROC of 0.56 and 0.33, respectively; **Suppl. Figure 3**). The varied performance across folds is most likely attributed to the well-known sensitivity of deep learning approaches to the quality of training data. In this case, we may speculate, that the testing samples from TEST1 group in folds 1 and 5 are not well represented by the training data. Although those differences were not investigated in presented experiment, we note it as a promising subject for separate XAI-based investigation. The errors were also noticeable in the calculated confusion matrix (average of 3.125 True Positives, 3.125 True Negatives, 0.375 False Positives and 0.75 False Negatives). Although this obstacle could not be tackled in this study due to limited sample size, we are positive that inclusion of more data on albinism will allow to mitigate this problem and enhance the performance of the trained CNN.

Surprisingly, despite considerable errors demonstrated on the TEST1, the results on the TEST2 demonstrated a perfect retrieval of single PWA sample in the data, as well as a marginal fraction of False Positives (0.6 vs 209.4 True Negatives). While this result requires further validation, ideally done with larger sample of training data and even higher-class imbalance in the testing sample, we consider this result to be highly encouraging for future attempts.

MSE per image					
Data		Architecture of encoder			
		Layers: 1<br>Initial feature maps: 2<br>Scaling factor: 2	Layers: 1<br>Initial feature maps: 4<br>Scaling factor: 1	Layers: 2<br>Initial feature maps: 32<br>Scaling factor: 1	Layers: 1<br>Initial feature maps: 8<br>Scaling factor: 2
Dev-test					
ABIDE (n=36))	control	17.1	20.4	25.0	27.8
Athletes (n=5)	control	9.7	11.6	14.2	16.8
HCP (n=107)	control	11.9	13.1	18.6	21.0
COBRE (n=6)	control	14.1	13.6	18.6	20.3
Leipzig (n=14)	control	28.2	31.2	38.1	41.2
MCIC (n=3)	control	21.0	22.6	32.2	33.3
CHIASM	control (n=1)	47.1	69.6	65.2	66.8
	albinism (n=1)	33.2	48.6	45.7	45.6
Ather et al.	control (n=3)	15.6	16.0	20.5	23.2
	albinism (n=3)	23.8	26.3	30.7	33.5
Test					
CHIASM	control (n=9)	48.4	72.0	67.5	71.3
	albinism (n=8)	39.8	62.6	54.3	57.9

Ather et al.	control (n=17)	25.8	27.4	33.1	36.5
	albinism (n=20)	26.0	27.7	33.2	36.0

Suppl. Table 4. Average Mean Square Error per image between original and re-encoded images.

The columns correspond to different architectures of auto-encoder used to encode and decode input patches from MRI images. The rows are grouped into dev-test and test groups, with each row corresponding to a unique combination of dataset and investigated cohort (control/albinism). The provided values were obtained by calculation of average MSE per image between original and re-encoded MRI patches containing optic chiasm.

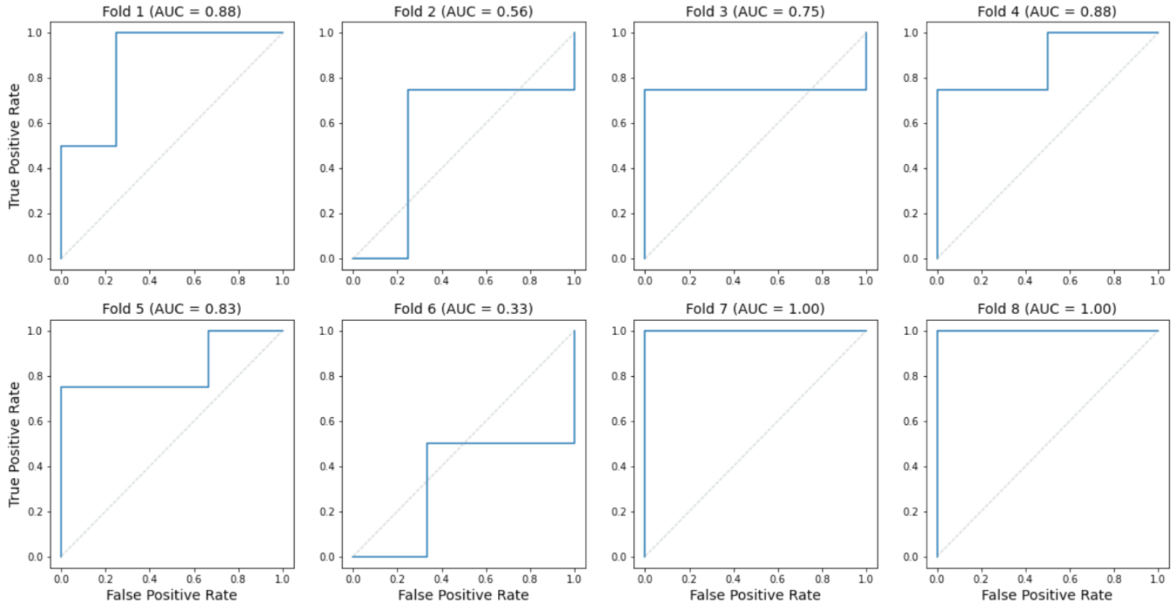

Suppl. Fig. 3. Performance of CHIASM-Net demonstrated with ROC curves across 8 folds. X-axis corresponds to False Positive Rate, Y-axis to True Positive Rate.

Supplement 9: Overfitting

As indicated in the **Discussion**, for an accurate evaluation of the performance of neural networks, it is necessary to consider the possibility of overfitting during training and the impact on a model's predictions and consequently its performance. In the present case, this inherent risk might be further enhanced due to the scarcity of training data (due to rarity of albinism) and reduced-size input-image patches. To mitigate the risk of overfitting the following means were taken:

- Increase of data sample: We placed high emphasis on collecting as large data sample as possible. For the PWA, which was most critical, we gathered the biggest MRI dataset on albinism that was until now reported in the literature. For the control data, we combined MRI data from multiple sources to ensure that the data well represented the distribution of configurations of normal optic chiasms.
- Limiting the number of parameters for model: Through reduction of depth of the feature extraction module (as compared to the original 3D U-Net) we limited the number of parameters learned during training. As such, the decision of using more shallow architecture instead of original may be considered an explicit regularization technique to reduce overfitting.
- Data augmentation: Prior to training, the input data were subjected to a random rigid transformation, such as translation and rotation. This technique allows for the generation of valid samples that were unseen by the model. Known as data augmentation [57], this is one of the implicit regularization methods used to reduce overfitting.
- Early stopping criterion: Each training session was prematurely stopped, if for an arbitrary number of epochs the DEV_TRAIN loss function did not improve. The final weights were selected from the epoch with the best DEV_TRAIN loss. This early stopping criterion technique allowed us to select weights that are not affected by prolonged overfitting.
- Multiple experiments: Finally, we employed an 8-fold validation, which allowed us to investigate overfitting on the sample of 8 separate training processes.

Suppl. Figure 4 demonstrates the learning curves recorded during training of the classification module, with each row corresponding to a single run of 8-fold validation. Prior to the discussion of overfitting, three essential points should be considered:

- The training curves recorded for DEV_TRAIN and TEST1 groups are strongly affected by noise. The cause of this is the very limited sample size of both groups (each containing 4 PWA and 4 controls)
- Records of training curves for TEST1 group were strictly prohibited from being accessed (visualization included) until the final model has been selected. This ensured that the decision about the final model was informed only by performance on TRAIN data (as in real world) and was not affectd by knowledge of the results on the test data.
- This training procedure had a maximum of 8000 epochs, with an early stopping criterion of 1000 epochs. Consequently, when inspecting the figures it is necessary to be aware that the selected optimal weights came from the training run that occured 1000 epochs before the end of training (see dashed blue line in **Suppl. Figure 4)**.

Below is a description of how we evaluated the recorded loss functions for each fold:

- Fold 1 – while the samples from DEV_TRAIN are well represented in TRAIN group, this is not the case for TEST1 sample. Consequently, the recorded loss and accuracy for TEST1 is visibly worse than the one for DEV_TRAIN, although analysis of TRAIN and DEV_TRAIN alone does not indicate no such issues, nor overfitting.
- Fold 2 – analogical case to fold 1. Although overfitting is not present, the TEST1 sample is not well represented in the training data.
- Fold 3 – the recorded loss for DEV_TRAIN demonstrates clear overfitting. Consequently, due to employed early stopping criterion the selected optimal weights come from one of the very first epochs, before the weights became overfitted.
- Fold 4 – analogical case to folds 1 and 2.
- Folds 5 and 6 - analogical case to fold 3.
- Fold 7 and 8 – representative desired case where TRAIN group well represents samples presented in DEV_TRAIN, as well as TEST1. Accordingly, the captured optimal weights ensure optimal performance of the network on unseen samples, as indicated by optimal ROC curves obtained for folds 7 and 8 (**Suppl. Fig. 3**).

In summary, although the risk of overfitting was increased by (i) small PWA sample and (b) the reduced size of the input patch, this was observed in only 3 out of 8 folds. Importantly, the application of the early stopping criterion prevented the selection of weights affected by overfitting for the final model. At the same time, we note the critical importance of the training data set's capability to represent all possible real-world cases. Even in the case of limited

sample of PWA images, in 2 out of 8 folds we were already able to observe the very strong performance of the trained model, if the above criterion is fulfilled.

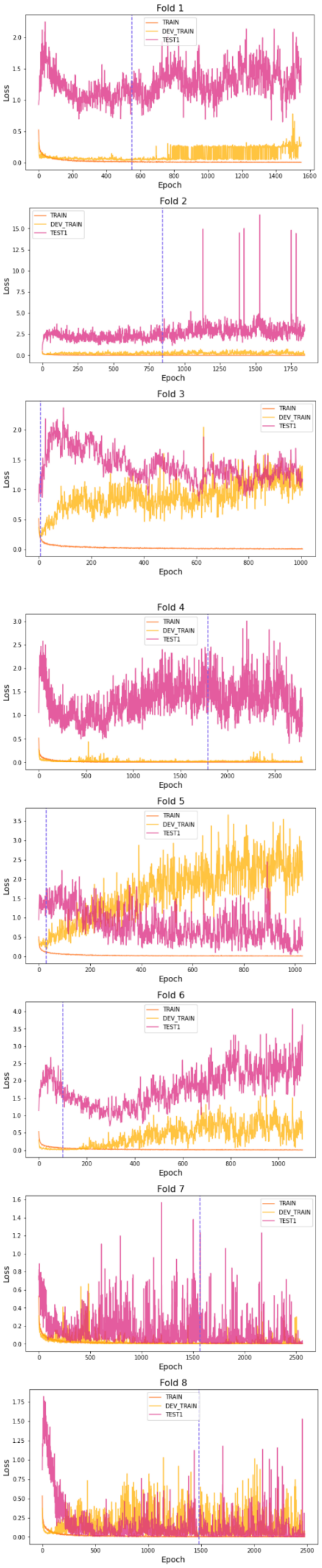
Fold 1
Fold 2
Fold 3
Fold 4
Fold 5
Fold 6
Fold 7
Fold 8
TRAIN
DEV_TRAIN
TEST1
Loss
Epoch

Suppl. Figure 4. Loss functions recorded over 8 separate runs of classification module training. Each row depicts a separate training run (as a part of 8-fold validation), the y-axis demonstrates the recorded loss function (Binary Cross Entropy), the x-axis indicates the epoch number. The 3 different colours depict separate groups - TRAIN (orange colour), DEV_TRAIN (yellow colour) and TEST1 (magenta colour). The dashed blue line indicates the epoch with best recorded values of DEV_TRAIN loss and which weights were deemed optimal and used for model evaluation.
